# Calcium and the Ca-ATPase SPCA1 modulate plasma membrane abundance of ZIP8 and ZIP14 to regulate Mn(II) uptake in brain microvascular endothelial cells

**DOI:** 10.1016/j.jbc.2022.102211

**Published:** 2022-07-02

**Authors:** Brittany L. Steimle, Danielle K. Bailey, Frances M. Smith, Shaina L. Rosenblum, Daniel J. Kosman

**Affiliations:** Department of Biochemistry, State University of New York at Buffalo, Jacobs School of Medicine and Biomedical Sciences, Buffalo, New York, USA

**Keywords:** blood–brain barrier, human brain microvascular endothelial cells, manganese transport, ZIP8, *SLC39A8*, ZIP14, *SLC39A14*, SPCA1, *ATP2C1*, calcium, BBB, blood–brain barrier, hBMVEC, human brain microvascular endothelial cell, IF, immunofluorescence, Mn, manganese, OGB1, Oregon Green BAPTA-AM, SPCA1, secretory pathway Ca^2+^ ATPase1, ZIP8, ZRT IRT-like protein 8, ZIP14, ZRT IRT-like protein 14, ZnT10, zinc transporter 10

## Abstract

Manganese (II) accumulation in human brain microvascular endothelial cells is mediated by the metal-ion transporters ZRT IRT-like protein 8 (ZIP8) and ZRT IRT-like protein 14 (ZIP14). The plasma membrane occupancy of ZIP14, in particular, is increased in cells treated with Mn^2+^, lipopolysaccharide, or IL-6, but the mechanism of this regulation has not been elucidated. The calcium-transporting type 2C member 1 ATPase, SPCA1, is a Golgi-localized Ca^2+^-uptake transporter thought to support Golgi uptake of Mn^2+^ also. Here, we show using surface protein biotinylation, indirect immunofluorescence, and GFP-tagged proteins that cytoplasmic Ca^2+^ regulates ZIP8- and ZIP14-mediated manganese accumulation in human brain microvascular endothelial cells by increasing the plasma membrane localization of these transporters. We demonstrate that RNAi knockdown of SPCA1 expression results in an increase in cytoplasmic Ca^2+^ levels. In turn, we found increased cytoplasmic Ca^2+^ enhances membrane-localized ZIP8 and ZIP14 and a subsequent increase in ^54^Mn^2+^ uptake. Furthermore, overexpression of WT SPCA1 or a gain-of-function mutant resulted in a decrease in cytoplasmic Ca^2+^ and ^54^Mn^2+^ accumulation. While addition of Ca^2+^ positively regulated ZIP-mediated ^54^Mn^2+^ uptake, we show chelation of Ca^2+^ diminished manganese transport. In conclusion, the modulation of ZIP8 and ZIP14 membrane cycling by cytoplasmic calcium is a novel finding and provides new insight into the regulation of the uptake of Mn^2+^ and other divalent metal ions–mediated ZIP metal transporters.

The brain utilizes manganese in many specialized functions. Some of these processes include assisting as a cofactor for the enzymatic reaction of glutamate conversion to glutamine *via* glutamine synthetase in astrocytes, as well as influencing the electrophysiological activity of glutamatergic neurons when released into the synaptic cleft (1). Under conditions where manganese levels are not properly managed in the brain, manganese is deposited in the basal ganglia (2). This causes neurodegeneration *via* altering neuronal activity through inhibition of voltage-dependent calcium channels and acting as a prooxidant (3). Therefore, the amount of manganese that crosses the blood–brain barrier (BBB) must be tightly regulated.

The P-type ATPase secretory pathway Ca^2+^ ATPase1 (SPCA1) has been reported to decrease cellular Mn^2+^ burden by aiding in Mn^2+^ efflux (4). SPCA1, encoded by the *ATP2C1* gene, is a Golgi-residing protein that pumps Ca^2+^ from the cytosol into the Golgi lumen to be packaged into vesicles and transported in the secretory pathway (5). Mutations in the *ATP2C1* gene are reported in patients with Hailey-Hailey disease, a rare skin disorder that is inherited in an autosomal dominant pattern (6). SPCA1 has also been implicated in Mn^2+^ import into the Golgi; the yeast ortholog PMR1 and SPCA1 in *C. elegans* were shown to transport Mn^2+^ with high affinity (7). A gain-of-function SPCA1 mutant, SPCA1(Q747A), when transfected into HeLa cells enhanced Mn efflux and cell viability following Mn treatment compared to cells expressing the wildtype protein (4). Based on this result, a model was proposed in which SPCA1 pumped Mn^2+^ in the Golgi following which the metal was secreted from the cell *via* anterograde vesicular trafficking. One precedent for this model is the trafficking of post-Golgi vesicles containing copper due to the action of the Cu-ATPase, ATP7A, that results in the export of cell copper (8, 9, 10, 11).

More recently, however, the product of the *SLC30A10* gene, zinc transporter 10 (ZnT10), has been explicitly identified as the essential Mn^2+^ efflux transporter (12, 13, 14). Loss of function mutations in ZnT10 consistently are linked to Mn-accumulation in the basal ganglia, and gene SNPs are commonly mapped in patients presenting with blood and brain excess (14, 15, 16). In addition, ZnT10 is essential to the excretion of manganese into the bile to maintain whole body Mn-homeostasis (12). While these findings do not negate the role proposed for SPCA1 in cell Mn-trafficking, they do suggest that the role played by this Ca^2+^-pump is more nuanced. Given the widely disparate ionic radii of Ca^2+^ and Mn^2+^ (17), their differential hydration (18), hydration energies (19), coordination number, and ligand field (20), one reasonable model is that SPCA1 is relatively nonselective and therefore not likely to play a specific role in Mn-homeostasis (21). On the other hand, it could play an indirect role due to its modulation of cytoplasmic and Golgi Ca^2+^. In this model, the abundance or activity of Mn^2+^ uptake and/or efflux transporters would be regulated by cytoplasmic Ca^2+^ and thus indirectly reliant on SPCA1 activity. The results described herein lend support to this premise.

The role of SPCA1 or ZnT10 in the transcellular trafficking of Mn^2+^ in brain microvascular endothelial cells (BMVECs) has not been examined. hBMVECs constitute the primary, impermeable barrier to free diffusion of systemic solutes into the brain’s interstitial space (22, 23). Thus, the expression and cell locale of the uptake and efflux transporters that support the flux of essential yet cytotoxic divalent metal ions at the BBB play a key role in cerebral metal-ion homeostasis. In a previous report, we demonstrated that the plasma membrane localization of the solute carriers ZRT IRT-like protein 8 (ZIP8) and ZRT IRT-like protein 14 (ZIP14) modulated the uptake of Mn^2+^ at both the apical, ‘blood’ and basal ‘brain’ side of an hBMVEC transwell BBB model (24). ZIP14 at the basal membrane, in particular, was essential to managing the hBMVEC retrieval of interstitial Mn^2+^ for efflux *back into circulation*. This *in vitro* finding provided a molecular explanation for the cerebral retention of manganese in the ZIP14 knock-out mouse (25, 26). These *in vivo* and complementary *in vitro* findings indicated that ZIP14 played a key role in managing brain manganese homeostasis.

ZIP8 and ZIP14 expression is positively regulated by inflammatory signals, *e.g*., by lipopolysaccharide (LPS) or IL-6 (24, 27, 28, 29). In the work noted above (24), the plasma membrane localization of ZIP14, in particular, increased following such treatments *independent* of an increase in protein expression. This observation suggested a model in which the trafficking of these solute transporters was downstream of a cytokine-initiated signal cascade; certainly, Ca^2+^ could be a candidate messenger of that signal. In this model, we considered a possible link to the cytoplasmic-Golgi Ca^2+^ balance mediated by SPCA1 and the Mn-related phenotypes linked to this Ca-ATPase. Here, we provide evidence that at least in part the link between SPCA1 function and cell Mn homeostasis is a role played by Ca^2+^ in the plasma membrane localization of ZIP8 and ZIP14. Specifically, cytoplasmic Ca^2+^ potentiates the functional localization of both solute transporters to increase Mn^2+^-uptake. In contrast, SPCA1-dependent Golgi uptake of Ca^2+^ or chelation of cytoplasmic Ca^2+^ knocks down Mn^2+^ uptake downstream of reduced plasma membrane localization of these transporters.

## Results

### The secretory pathway SPCA1 is expressed in hBMVECs

**Figure 1:**
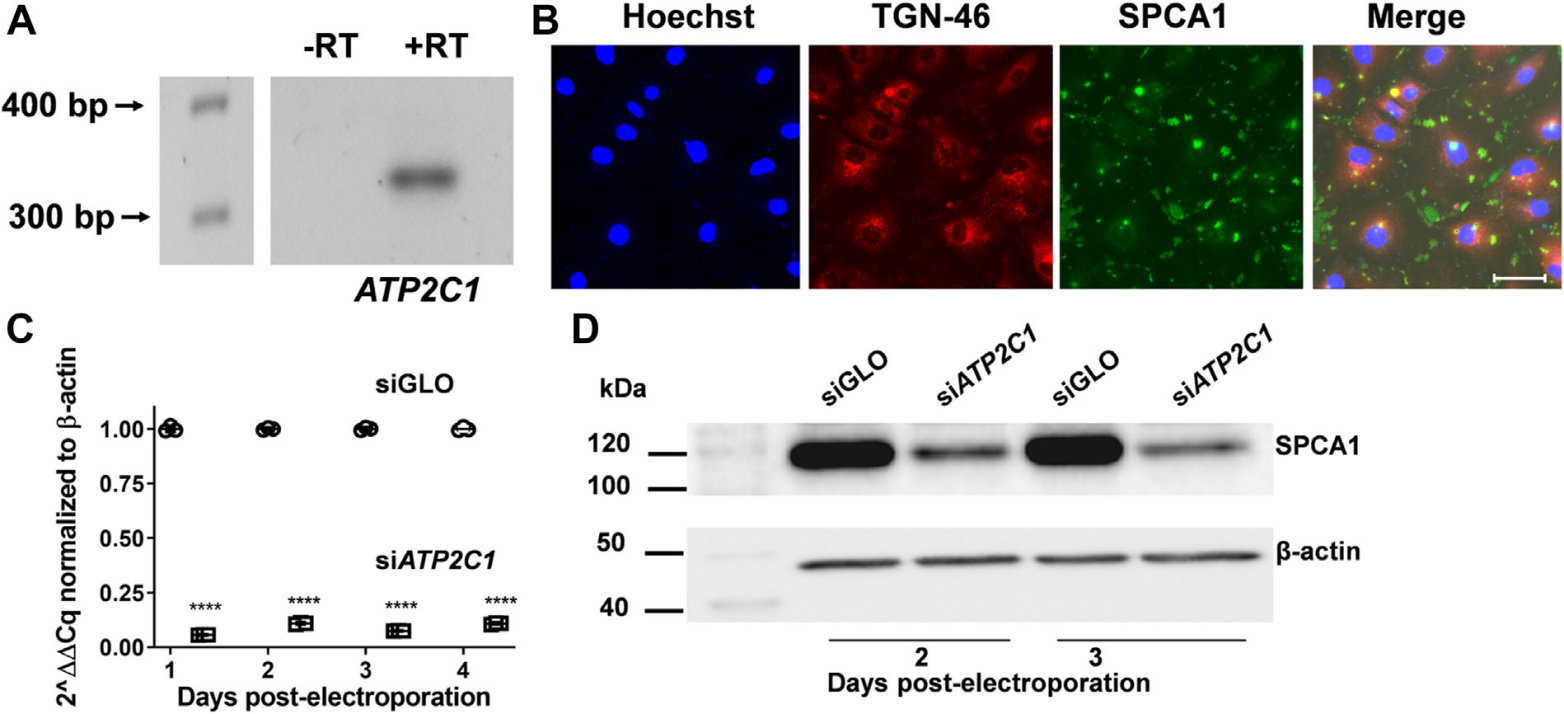
***ATP2C1* is expressed in hBMVECs and is subcellularly localized in the TGN**. *A*, transcript expression was confirmed by RT-PCR. RNA was collected, reverse-transcribed, and the resulting cDNA was run on a 2% agarose gel. -RT and +RT indicate the absence or presence of RTase to control for gDNA contamination. The predicted product size of *ATP2C1* with the primers listed in the Materials and Methods is 325 BP. *B*, endogenous SPCA1 in hBMVEC visualized by indirect immunofluorescence. Cells were grown on sterile coverslips to 75 to 80% confluency, fixed, blocked, and incubated overnight with primary antibodies to SPCA1 and TGN-46 Alexa Fluor 647. Coverslips were incubated in α-mouse Alexa Fluor 488-conjugated secondary for 1 h followed by a 10 min Hoechst 33342 nuclear stain. Coverslips were mounted using Prolong Gold mounting media and sealed. Images were acquired at 63X magnification with oil immersion on a Leica TCS SP8 confocal microscope. The white scale bar on the merged image represents a distance of 42.3 μm. *C*, transcript abundance of *ATP2C1* was quantified by qPCR. RNA was collected days 1 to 4 postelectroporation of siGLO (negative control) and siATP2C1, reverse-transcribed, and resulting cDNA was quantified by qPCR. The relative fold expression of *ATP2C1* presented was calculated by the standard ΔΔC_t_ method. Statistical significance was tested by *t* test analysis, ∗∗∗∗*p* < 0.0001. *D*, SPCA1 protein is reduced upon electroporation with siATP2C1. Cell lysates were collected from cells electroporated with siGLO or siATP2C1 2 to 3 days postelectroporation. Lysates were subject to Western blotting and probed for α-SPCA1 and β-actin (loading control). hBMVEC, human brain microvascular endothelial cell; SPCA1, secretory pathway Ca^2+^ ATPase1.

The expression of the secretory pathway SPCA1 was assessed at the transcript level. hBMVEC RNA was extracted, reverse-transcribed, the resulting *ATP2C1* gene fragment PCR amplified, and the PCR product was analyzed on an agarose gel (Fig. 1*A*). No product was obtained in the absence of initial reverse-transcription. Expression and localization of SPCA1 in hBMVECs were verified by indirect immunofluorescence. As shown in Figure 1*B*, SPCA1 was found to be intracellularly localized, partially residing in the trans-Golgi as marginal co-localization with TGN-46 is observed. In the HeLa cell cervical cancer cell line, N2A neuroblastoma cells, human aortic smooth muscle cells, and mouse TM4 testis cells, SPCA1 was found to be present in the cis-Golgi and trans-Golgi, as well as in late endosomes (30, 31, 32).

### siRNA knockdown of ATP2C1 suggests a role in Mn^2+^ accumulation, but not in Mn^2+^ efflux in hBMVECs

There are various reports of SPCA1 as a dual-functioning protein that pumps both Ca^2+^ and Mn^2+^ from the cytosol into the Golgi, cargo that then is exported *via* the secretory pathway (4, 33, 34). Many studies have reported on Ca^2+^ transport by SPCA1, but the role of SPCA1 in intracellular Mn^2+^ transport has received considerably less attention. Therefore, we sought to interrogate the role that SPCA1 played in Mn-trafficking in hBMVECs by knocking down the *ATP2C1* transcript with targeted siRNAs.

Transcript levels of ATP2C1 were examined by qPCR 1 to 4 days post electroporation of hBMVECs with siRNAs. Knockdown efficiency of ATP2C1 remained at 89 to 94% up to 4 days post electroporation (Fig. 1*C*). SPCA1 protein abundance was examined in lysates taken from these cells. SPCA1 protein level was decreased by 79% and 87% 2 and 3 days post electroporation, respectively (Fig. 1*D*).

**Figure 2:**
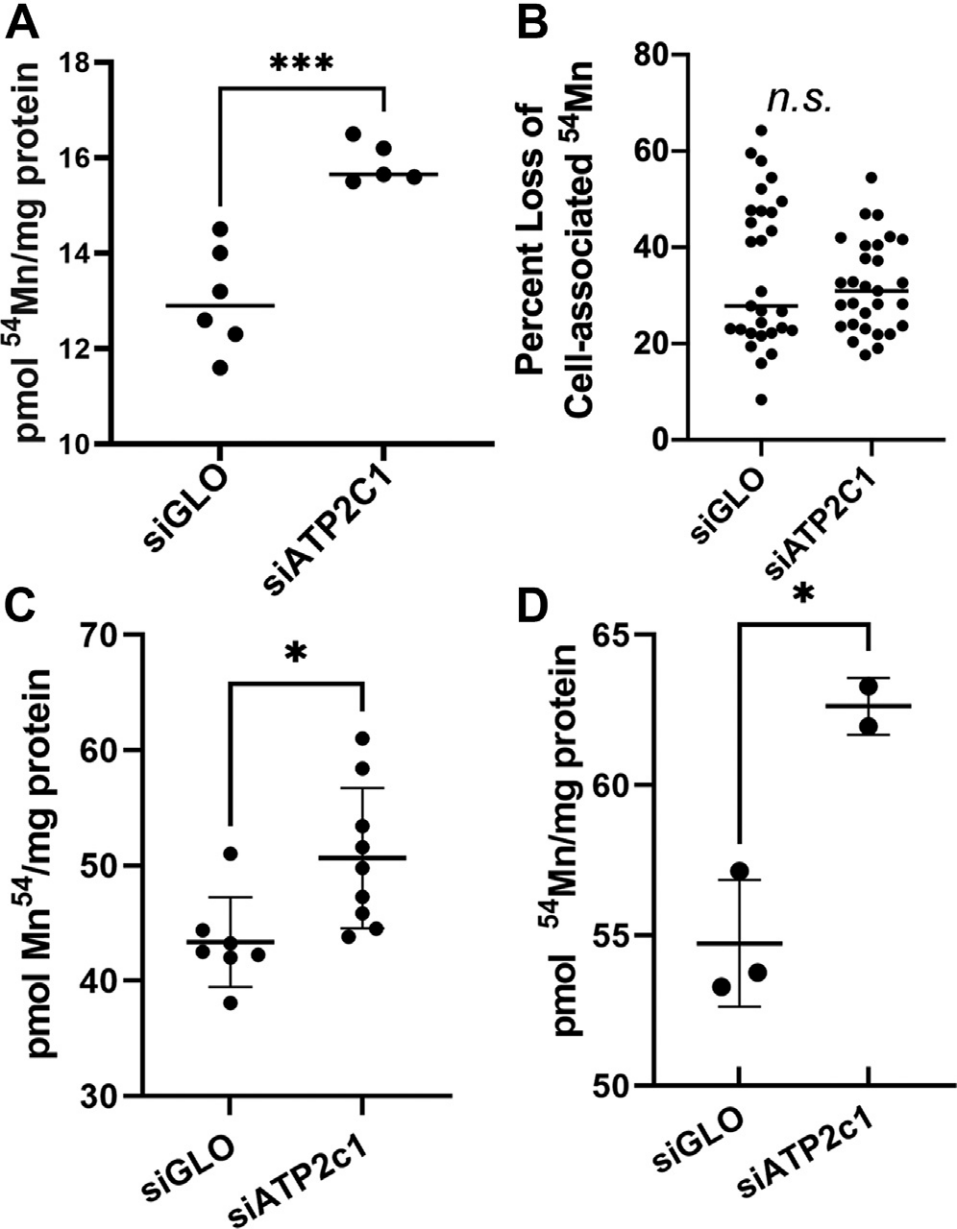
**Knocking down *ATP2C1 via* siRNA augments Mn**^**2+**^**accumulation, but not efflux in hBMVECs.***A*, ^54^Mn^2+^ accumulation is enhanced in siATP2C1 hBMVEC. hBMVECs grown in monolayers were loaded with 200 nM ^54^Mn^2+^ for 3 h. At least five experimental trials replicated this result. Statistical significance was tested by *t* test analysis, ∗∗∗*p* < 0.001. *B*, ^54^Mn^2+^ efflux is not altered in siATP2C1 hBMVEC. hBMVEC-grown monolayers were loaded with 200 nM ^54^Mn^2+^ for 18 h, followed by a 3 h efflux period. Percent loss of ^54^Mn from hBMVEC is reported, normalized to protein content. This panel combines data taken from five separate trials. Statistical significance was tested by *t* test analysis, n.s. = not statistically significant. siATP2C1 knocks down ^54^Mn accumulation at both the apical (*C*) and basolateral hBMVEC membranes (*D*) in a transwell model system. hBMVECs grown in transwells were loaded with 200 nM ^54^Mn^2+^ for 24 h apically (*C*) or basally (*D*), lysed for gamma counting, and normalized to total protein content. At least seven experimental replicates for two separate trials (*C*) and two experimental replicates (*D*) are illustrated. Statistical significance was tested by *t* test analysis, ∗*p* < 0.05. hBMVEC, human brain microvascular endothelial cell.

^54^Mn^2+^ uptake and efflux were quantified in these SPCA1-knockdown hBMVEC, comparing these values to those quantified in the siGLO control transfectants. A first set of experiments were conducted in monolayers in standard 24-well plates. In 3 h ^54^Mn^2+^ loading, knockdown cells accumulated ∼25% more ^54^Mn^2+^ than the corresponding siGLO negative control (Fig. 2*A*). Note that ^54^Mn accumulation is linear from 0 to 3 h; thus, these 3-h time-point values are a reflection of uptake and not steady-state radionuclide accumulation (24). In contrast, there was no difference in the ^54^Mn-efflux from control in comparison to si*ATP2C1*-transfected hBMVEC (Fig. 2*B*). In this efflux experiment, si*ATP2C1* and control cells were loaded with ^54^Mn^2+^ for 18 h, followed by a 3 h efflux period with the data expressed as the percent accumulated ^54^Mn lost in this period of efflux. These data suggest that in hBMVECs, SPCA1 plays little if any role in Mn-efflux.

^54^Mn hBMVEC uptake assays were conducted also in a transwell format as described previously (24). In this experiment, cell ^54^Mn accumulation was quantified after addition of the radionuclide to either the apical (Fig. 2*C*) or basal (bottom) chamber (Fig. 2*D*). The data show that as in 3 h uptake in monolayers, knockdown of *ATP2C1* results in an increase in ^54^Mn accumulation irrespective of the directionality of the uptake process. However, the increase is greater with respect to accumulation at the apical (16.8%) in comparison to the basolateral membrane (14.4%). While this behavior will be discussed in more detail in the Discussion, note also the more robust basal ^54^Mn uptake, a feature that we have linked to the abundance of basolateral ZIP14 found in these polarized cells as quantified by surface protein biotinylation (24).

### Overexpression of SPCA1 and Q747A hyperactive mutant reduces Mn^2+^ uptake in hBMVECs

Mukhopadhyay and Linstedt studied the impact of a predicted gain-of-function mutant, SPCA1(Q747A), on Mn^2+^ cytotoxicity (4). The gain-of-function mutant was predicted to have an enhanced ion permeation cavity, thereby increasing the ability of the ATPase to clear cytoplasmic cations, whether Ca^2+^ or Mn^2+^. Overexpression of this SPCA1 mutant in HeLa cells was able to increase Mn^2+^-transport in isolated Golgi membranes, decrease overall Mn-burden and decrease Mn-cytotoxicity (4).

**Figure 3:**
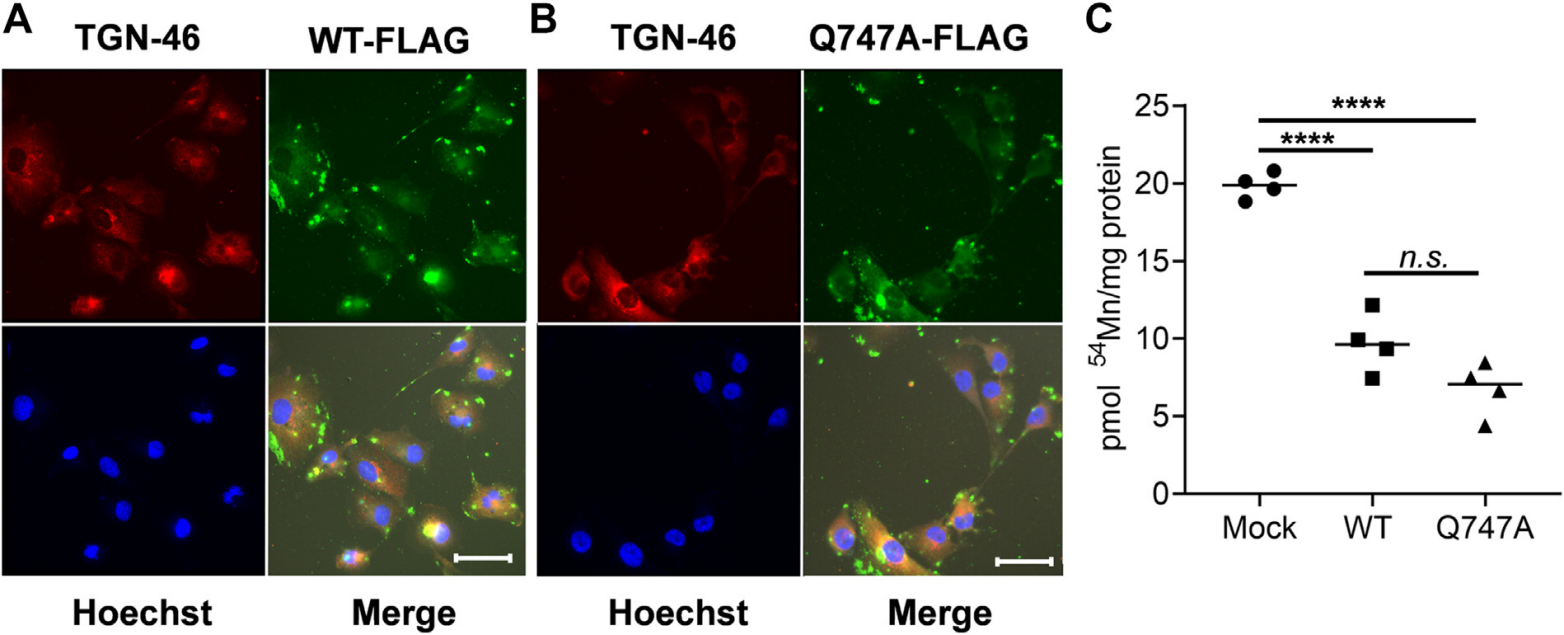
**Episomal expression of WT SPCA1-FLAG or Q747A SPCA1-FLAG reduces Mn**^**2+**^**accumulation in hBMVECs.***A* and *B*, WT SPCA1-FLAG and Q747A SPCA1-FLAG are intracellularly localized to the TGN. Transfected cells were grown on sterile coverslips to 50 to 60% confluency, fixed, blocked, and incubated overnight with primary antibodies to FLAG and TGN-46 Alexa Fluor 647. Coverslips were incubated in α-mouse Alexa Fluor 488-conjugated secondary for 1 h followed by a 10 min Hoechst 33342 nuclear stain. Coverslips were mounted using Prolong Gold mounting media and sealed. Images were acquired at 63X magnification with oil immersion on a Leica TCS SP8 confocal microscope. The white scale bars on the merged images represent a distance of 42.3 μm. *C*, transfection of WT SPCA1-FLAG and Q747A SPCA1-FLAG decreases ^54^Mn^2+^ accumulation. hBMVECs were loaded with 200 nM ^54^Mn^2+^ for 24 h. At least three experimental trials replicated these results shown in panel C. Statistical significance was tested by *t* test analysis, ∗∗∗∗*p* < 0.0001; n.s. = not statistically significant. hBMVEC, human brain microvascular endothelial cell; SPCA1, secretory pathway Ca^2+^ ATPase1.

To examine this behavior in hBMVECs, plasmids were constructed to overexpress WT SPCA1 or the hyperactive Q747A mutant; the proteins encoded by these constructs carried a FLAG tag to distinguish them from the endogenous protein. Expression of these WT and Q747A SPCA1 constructs was confirmed by indirect immunofluorescence using an α-FLAG antibody (Fig. 3, *A* and *B*, respectively). Since SPCA1 knockdown resulted in augmented Mn^2+^ accumulation in hBMVECs, we predicted that overexpression of WT or the SPCA1^UP^ allele would result in a decreased Mn^2+^ accumulation. To test this prediction, WT, Q747A SPCA1, or mock-transfected cells were loaded with ^54^Mn^2+^ for 24 h. As expected, overexpression of WT SPCA1 resulted in less Mn^2+^ accumulation than quantified in mock-transfected cells; the Q747A mutant induced an even greater decrease compared to both mock and WT SPCA1–transfected hBMVEC (Fig. 3*C*). Collectively, these results support the premise that SPCA1 activity negatively modulates Mn^2+^-uptake while playing no evident role in Mn-efflux in hBMVECs.

### Cytoplasmic Ca^2+^ managed by SPCA1 regulates Mn^2+^ accumulation in hBMVECs

**Figure 4:**
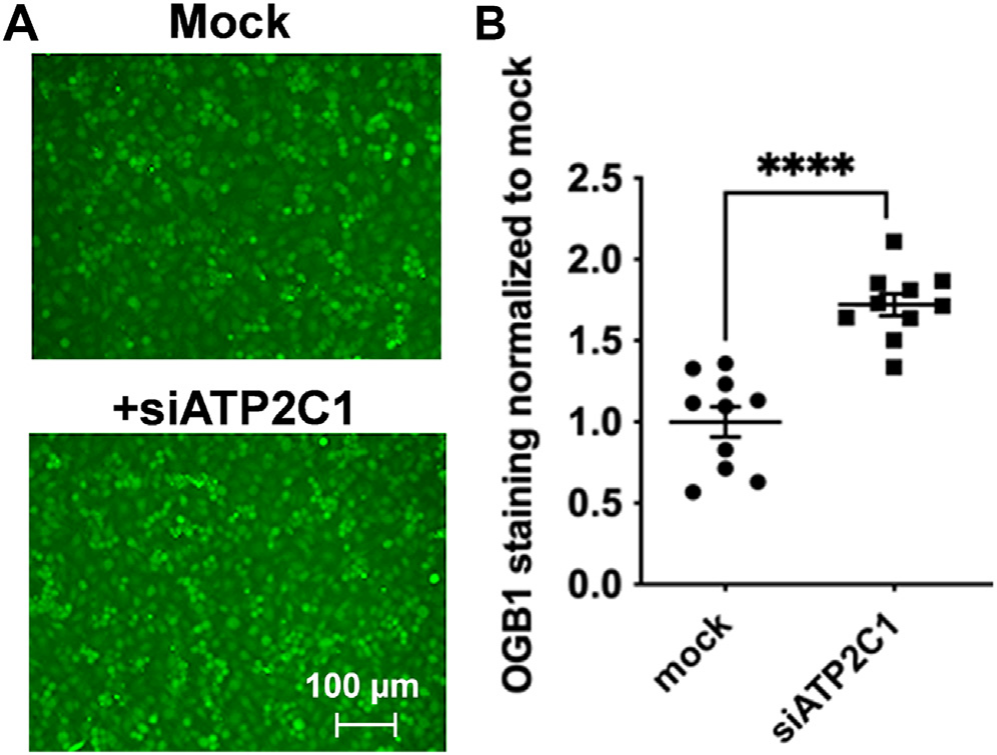
**SPCA1 manages intracellular Ca**^**2+**^**levels in hBMVECs.***A*, mock-transfected and siATP2C1 hBMVECs were stained with OGB1. Cells were grown to 70 to 80% confluency prior to OGB1 staining. 20X images were taken on a Bio-Rad ZOE imager. The scale bars indicate a distance of 100 μm. *B*, green fluorescence intensity was compared to no OGB1 stain control and quantified. n = 6/condition. Statistical significance was tested by *t* test analysis in comparison to untreated cells, ∗∗∗∗*p* < 0.0001. hBMVEC, human brain microvascular endothelial cell; SPCA1, secretory pathway Ca^2+^ ATPase1.

SPCA1 is well-established as a Golgi-localized, Ca^2+^-transporting ATPase pump (5, 30, 35). Thus, we investigated whether knocking down *ATP2C1* in hBMVECs altered chelatable, likely cytoplasmic Ca^2+^-levels. hBMVECs were treated with Oregon Green BAPTA-AM (OGB1), conjugated to Alexa 488, to obtain a semiquantitative measure of intracellular Ca^2+^ (Fig. 4*A*). There is a proportional relationship between the fluorescence intensity of this probe with the levels of intracellular Ca^2+^ (36). Furthermore, OGB1 is considered to be fairly specific for cytoplasmic calcium (37). Compared to mock-transfected cells, si*ATP2C1*-transfected cells had statistically more chelatable Ca^2+^ (Fig. 4*B*).

**Figure 5:**
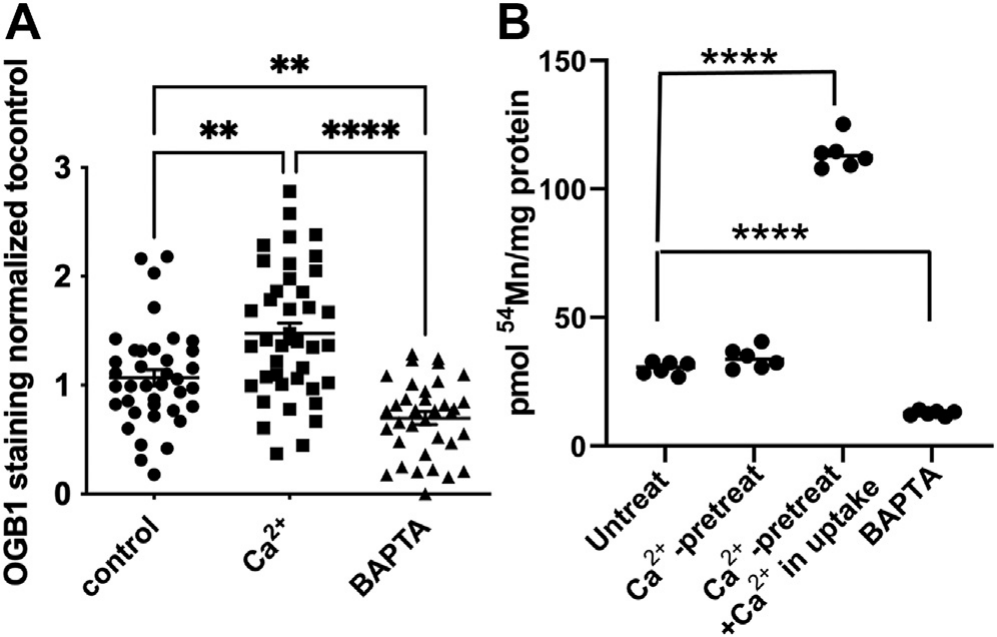
**Cytoplasmic Ca**^**2+**^**correlates positively with**^**54**^**Mn uptake.***A*, hBMVECs were grown in media without Ca^2+^ for 2 days, then treated with 2 mM CaCl_2_ or 10 μM BAPTA-AM for 1.5 h and imaged for intracellular Ca^2+^ levels by OGB1 staining. Quantification is a measure of OGB1 intensities from n = 8 biological replicates, with n ≥ 5 ROIs quantified for each image. Statistical significance was determined using one-way ANOVA and Tukey’s multiple comparison test, ∗∗*p* < 0.01, ∗∗∗∗*p* < 0.0001. *B*, levels of intracellular Ca^2+^ influence ^54^Mn^2+^ efflux in hBMVEC. hBMVECs were pretreated with 2 mM CaCl_2_ or 10 μM BAPTA-AM for 1.5 h prior to loading cells with 200 nM ^54^Mn^2+^ for 1 h in the presence or absence of 2 mM CaCl_2_. n = 6 per condition. Statistical significance was tested by *t* test analysis in comparison to untreated cells, ∗∗∗∗*p* < 0.0001. hBMVEC, human brain microvascular endothelial cell; SPCA1, secretory pathway Ca^2+^ ATPase1.

Thus, SPCA1 knockdown resulted in an increase in cytoplasmic Ca^2+^ and cell Mn^2+^. This pattern indicated that cytoplasmic Ca^2+^ may positively regulate Mn^2+^ accumulation in hBMVECs. To test this inference, hBMVECs were pretreated with Ca^2+^ or BAPTA-AM for 1.5 h, followed by imaging cytoplasmic Ca^2+^ using OGB1 and quantifying 1 h ^54^Mn^2+^ uptake in the presence or absence of CaCl_2_ in the loading media. As noted, ^54^Mn-accumulation by hBMVECs is linear from 0 to 3 h; thus, quantification of cell accumulation in a 1 h time period represents a kinetic measurement (24). As expected, Ca^2+^ treatment increased chelatable cytoplasmic Ca^2+^, whereas BAPTA-AM pretreatment attenuated it (Fig. 5*A*). ^54^Mn^2+^ uptake in BAPTA-AM–treated cells was knocked down by 50%, whereas CaCl_2_ pretreatment alone had no effect. However, CaCl_2_ pretreatment along with CaCl_2_ present in the loading media substantially augmented ^54^Mn^2+^ accumulation (Fig. 5*B*). These results support the premise that Mn^2+^ uptake by hBMVECs positively correlates with intracellular Ca^2+^.

### Intracellular Ca^2+^ regulates the surface presentation of ZIP8 and ZIP14

**Figure 6:**
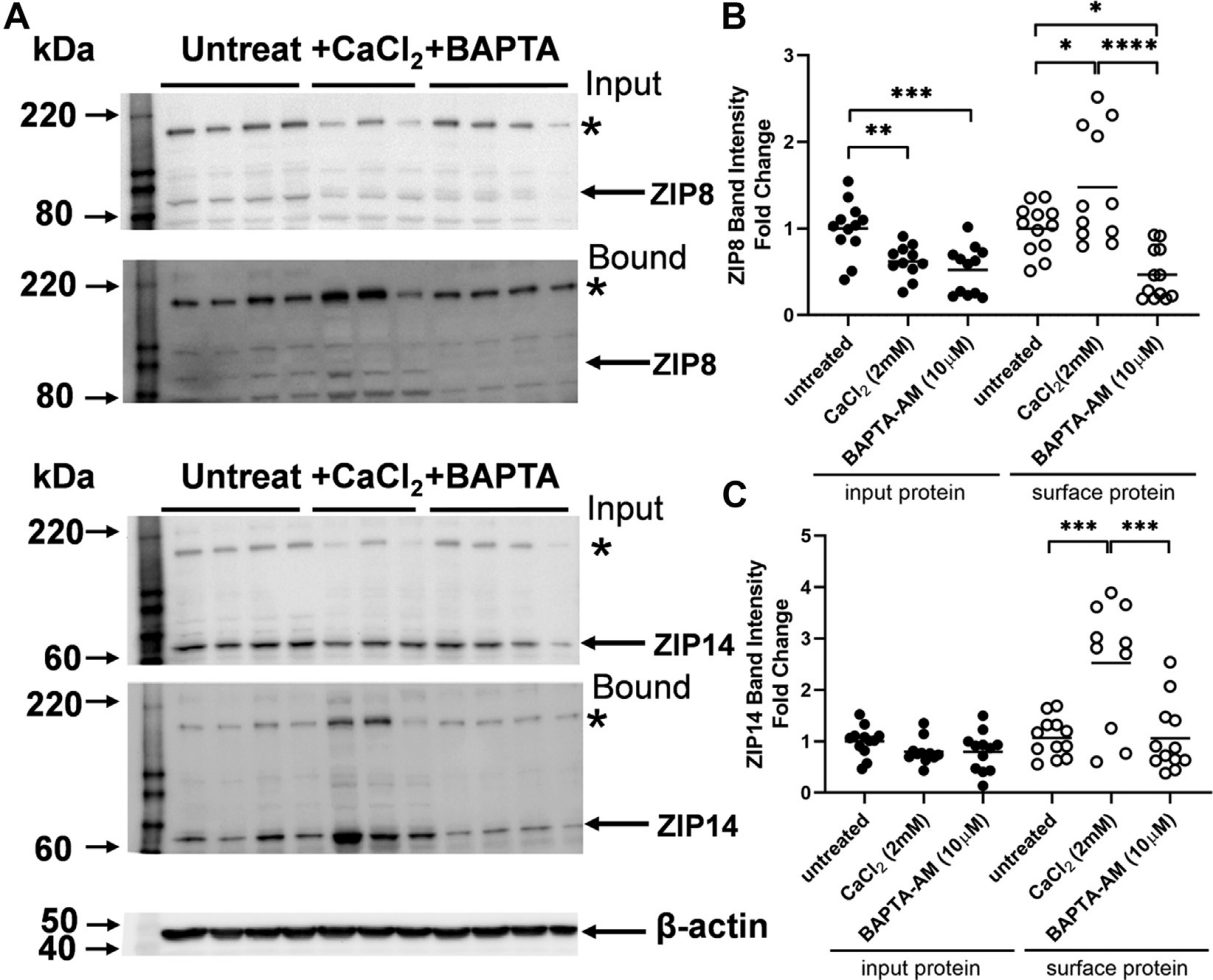
**CaCl**_**2**_**treatment increases cell surface residence of ZIP8 and ZIP14 in hBMVECs.***A*, cells were pretreated with 2 mM CaCl_2_ or 10 μM BAPTA-AM in RPMI 1640 minus Ca^2+^, minus serum supplemented media for 3 h prior to cell-surface biotinylation of hBMVEC for ZIP8 and ZIP14. *Arrows* represent the monomeric form of the protein, and *asterisks* designate an oligomeric form of the protein. Fold change in band intensity was normalized to β-actin probed in the blot of input (total) protein. The total and biotinylated cell-surface protein (bound) was quantified for ZIP8 (*B*) and ZIP14 (*C*); n = 3 to 4 biological replicates per condition were performed each with n = 4 experimental replicates. The blots in panel *A* are representative of experimental replicates from one of the labeling experiments. Note that in this analysis, following probing for ZIP8, the blots were stripped and then probed for ZIP14. Statistical significance was determined using one-way ANOVA and Tukey’s multiple comparison test for input and surface protein groups separately, ∗*p* < 0.05, ∗∗*p* < 0.01, ∗∗∗*p* < 0.001, ∗∗∗∗*p* < 0.0001. hBMVEC, human brain microvascular endothelial cell; ZIP8, ZRT IRT-like protein 8; ZIP14, ZRT IRT-like protein 14.

In a previous report, we showed that ZIP8 and ZIP14 supported the major fraction of Mn^2+^ uptake in hBMVECs. We demonstrated that the plasma membrane localization of ZIP14 increased upon Mn treatment and as a result of inhibition of dynamin-mediated endocytosis (24). In addition, the plasma membrane localization of ZIP8 is positively influenced by media iron (38). These patterns indicate these two divalent metal-ion transporters undergo anterograde and retrograde cycling; both contain retrieval signals linked to such behavior (39). The model that intracellular Ca^2+^ played a role in this cycling was tested by treating hBMVECs with Ca^2+^ or BAPTA-AM for 3 h and then using cell-surface biotinylation to quantify the plasma membrane occupancy of ZIP8 and ZIP14 (Fig. 6*A*). BAPTA-AM pretreatment did not alter total or surface ZIP8 or ZIP14 as detected by the biotinylation approach. On the other hand, Ca^2+^ treatment elevated both ZIP8 and ZIP14 plasma membrane residence (Fig. 6, *B* and *C*, respectively). The total amount of ZIP14 was unchanged in this 3-h Ca^2+^ treatment (Fig. 6*C*), but there was a significant decrease in total ZIP8 (Fig. 6*B*), specifically a loss of a multimeric form of ZIP8 (Fig. 6*A*, top). This likely is a dimer; experiments with recombinant ZIP proteins have demonstrated that both monomer and dimer forms are detected in denaturing gels like those used here (40).

### Calcium induces a perinuclear to trans-Golgi trafficking of ZIP8 in hBMVECs and ZIP14-GFP in HEK293 cells

**Figure 7:**
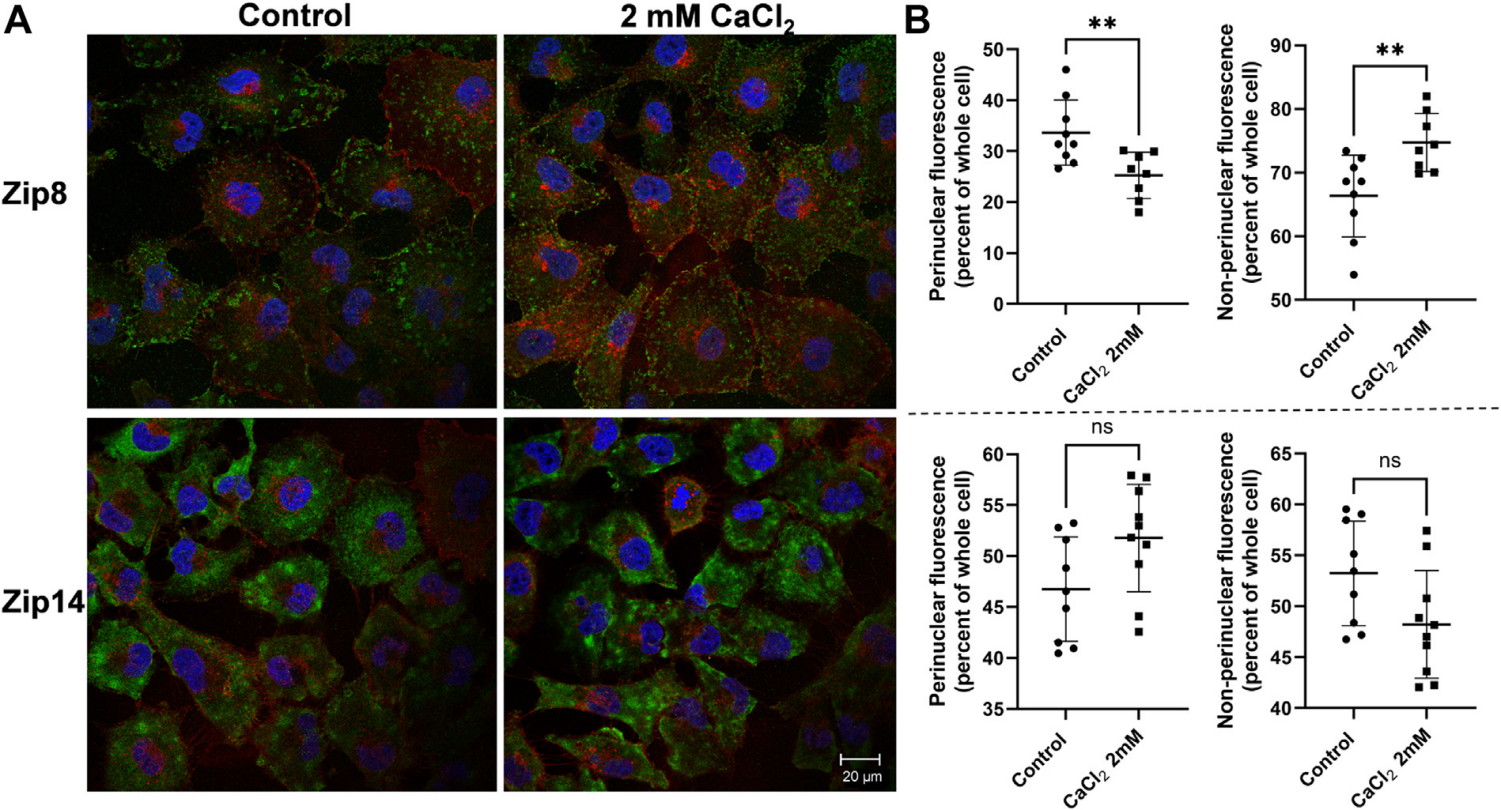
**Cytoplasmic Ca**^**2+**^**induces the trafficking of ZIP8.***A*, hBMVECs grown on coverslips were treated with either 2 mM CaCl_2_ or RPMI 1640 minus Ca^2+^, minus serum media as an untreated control. Cells were fixed, stained with wheat germ agglutinin (WGA), blocked, and then incubated with rabbit primary antibodies against ZIP8 and ZIP14. Coverslips were incubated in anti-rabbit Alexa Fluor 488-conjugated secondary for 1 h followed by a 10-min nuclear stain with Hoechst 33342. Coverslips were mounted using Prolong Gold antifade mounting media and sealed. Images were acquired at 63 × magnification with oil immersion on a Leica TCS SP8 confocal microscope. Images were adjusted for brightness and quantified using ImageJ. *B*, perinuclear fluorescence represents pixel values around the nucleus, and nonperinuclear fluorescence represents values obtained by subtracting perinuclear fluorescence from whole-cell fluorescence. Both perinuclear and nonperinuclear measurements were divided by whole-cell measurements to obtain the percent of whole-cell values. For ROI quantification, n = 10 cells in seven separate fields per condition were analyzed. Statistical significance was determined using compared to untreated control, ns = not statistically significant, ∗∗*p* < 0.01. ZIP8, ZRT IRT-like protein 8; ZIP14, ZRT IRT-like protein 14.

The relation between ZIP8 and ZIP14 plasma membrane localization was examined also by indirect immunofluorescence (Fig. 7*A*). While no specific changes in ZIP14 localization could be statistically quantified, there was a significant dispersal of ZIP8 from the perinuclear region to a more vesicular presentation (Fig. 7*B*). A similar pattern of ZIP8 re-localization from primarily intracellular to cell surface presentation was observed in HEK293 cells treated with iron (38). Note, however, that in hBMVECs, only a small fraction of total ZIP8 is in the plasma membrane compared to total cellular protein, a finding reported also in the A549 lung epithelial and HIBCPP choroid plexus papilloma cell lines (41, 42), and human proximal tubular epithelial cells (43). In addition, the pattern of ZIP8 and ZIP14 cell localization observed in these latter cells is equivalent to the results shown herein.

**Figure 8:**
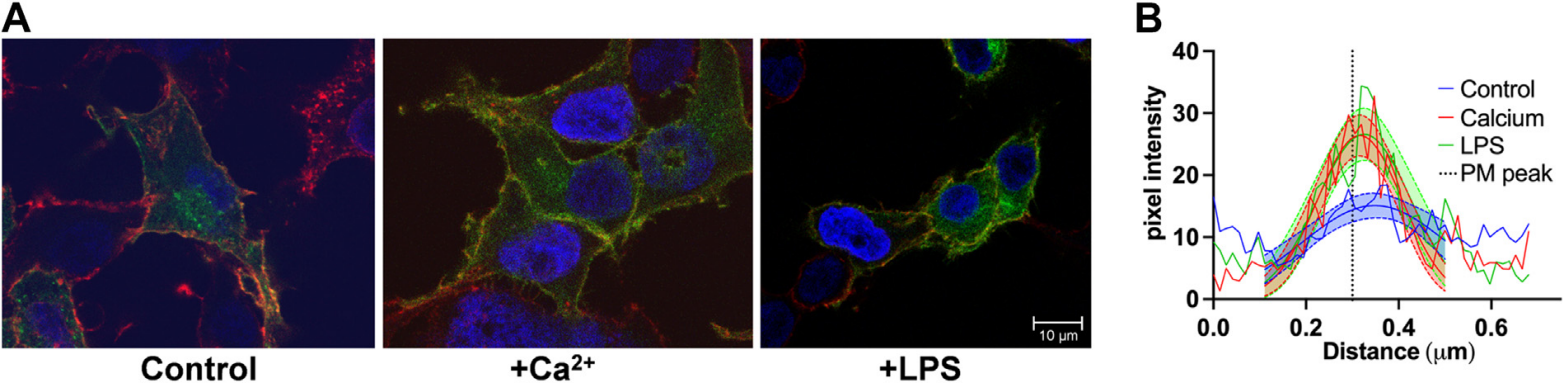
**Cytoplasmic Ca**^**2+**^**and LPS enhances the plasma membrane localization of ZIP14-EGFP.** HEK293T/17 cells transfected with either pEGFP-N1-ZIP14 or pEGFP-N1 as a vector control were grown on coverslips for 2 days in DMEM minus Ca^2+^, then treated for 2 h with either 2 mM CaCl_2_, 1 μg LPS, or DMEM minus Ca^2+^, minus serum media alone as a control. *A*, cells were fixed and stained with WGA and Hoechst. Images were acquired at 63X magnification with oil immersion on a Leica TCS SP8 confocal microscope. *B*, ZIP14-GFP peak at the plasma membrane was quantified as outlined in Methods. The resulting histograms for the plasma membrane peak ± 0.2 μm were subjected to non-linear regression fit to a Gaussian model, and the 95% confidence intervals were calculated and plotted. DMEM, Dulbecco's modified Eagle's medium; LPS, lipopolysaccharide; WGA, wheat germ agglutinin.

Transporter localization in response to cell Ca^2+^ was interrogated also by use of a carboxyl-terminal ZIP14-EGFP fusion protein episomally expressed downstream of the CMV promoter following treatment with either Ca^2+^ (2 mM) or LPS (1 μg/ml) for 2 h prior to cell imaging. Examples of the differential localization of ZIP14-EGFP in control, Ca^2+^-treated, or LPS-treated cells are shown in Figure 8*A*. By quantifying pixel density by an regions of interest (ROI) approach as described in Materials and Methods, an increase in membrane localization of the ZIP14 fusion protein was quantified. The result is shown in Figure 8*B* with the 95% confidence limits indicated. The best fit values for the pixel amplitudes for control, Ca^2+^-treated and LPS-treated samples were 15.1 ± 0.2, 26.4 ± 0.1, and 26.6 ± 0.1, respectively.

## Discussion

In a previous study, we reported that both ZIP8 and ZIP14 contribute to Mn^2+^ accumulation in a model blood–barrier cell line, hBMVEC (24); Scheiber *et al.* have demonstrated the same function for these two divalent metal-ion transporters in a lung carcinoma epithelial cell line, A549 (42). We also demonstrated that whereas ZIP8 was equally abundant in the apical (blood) and basolateral (abluminal) membranes of hBMVECs grown in transwells, 90% of plasma membrane–associated ZIP14 was found on the ‘brain’ side of this model BBB. A comparable asymmetric localization has been reported for these two transporters in the HIBCPP choroid plexus cell line (41). That these two transporters might contribute differentially to cellular manganese homeostasis was indicated also by differences in the regulation of their plasma membrane localization. While both transporters responded positively to LPS treatment, only ZIP14 membrane localization increased following treatment with dynasore and Mn^2+^ (24). The former observation suggests that the residence lifetime of ZIP14 in the plasma membrane is regulated by dynamin-dependent retrieval. The literature also shows that the steady-state plasma membrane abundance of either transporter represents only a small fraction of the cell total of either protein (24, 38, 41). Taken together, the data are consistent with the premise that the *efficacy* of these two manganese and iron transporters can be regulated by modulation of their anterograde and retrograde trafficking to and from the plasma membrane.

Here, we provide evidence that this membrane occupancy responds to intracellular (cytoplasmic) Ca^2+^ levels, and both cytoplasmic Ca^2+^ and transporter localization are responsive to SPCA1 activity. We show that *ATP2C1*/SPCA1 is expressed in hBMVECs. Reducing SPCA1 protein abundance by siRNA knockdown increases chelator-accessible cell Ca^2+^ and that increase is mirrored by an increase in ^54^Mn accumulation; conversely, overexpressing WT or hyperactive mutant Q747A SPCA1 reduced both quantities. A key finding was that modulation of SPCA1 expression modulated Mn^2+^ uptake exclusively with no effect on Mn^2+^ efflux.

Note, however, that while CaCl_2_ pretreatment alone did not alter ^54^Mn uptake (Ca-free media used in the transport assay), supplementing the uptake media with CaCl_2_ amplified ^54^Mn uptake over 2-fold. One possible interpretation of this observation is that Ca^2+^ activates ZIP-dependent Mn-uptake; a similar model holds for Ca^2+^ potentiation of ferroportin-dependent iron efflux (44). More likely, however, is in the washing of the Ca^2+^-treated cells and re-equilibration for the subsequent ^54^Mn-uptake, the intracellular Ca^2+^ and transporter localization returned to baseline levels. In support of this premise, as quantified by protein biotinylation, surface ZIP8 and ZIP14 were increased ∼2-fold when hBMVECs were treated with CaCl_2_. This is consistent with the fact that Mn^2+^ uptake increased ∼3-fold with Ca^2+^ treatment.

While indirect immunofluorescence could not replicate these biochemical data quantitatively, they did reveal a general anterograde trafficking of ZIP8 upon Ca^2+^ treatment. A similar behavior was found for the ZIP14-GFP fusion. Taken together, the data support the proposal that as is the case with other metabolite signals, *e.g.,* Zn^2+^ and Fe^2+^, Ca^2+^ also modulates the cellular distribution of ZIP8 and ZIP14.

That this putative Ca^2+^ signal is sensed by both ZIP8 and ZIP14 is indicated by the ^54^Mn accumulation pattern demonstrated in the transwell cell platform. As noted above, previous work demonstrated that while ^54^Mn uptake at the apical surface was mediated equivalently by the two transporters, ZIP14 contributed ∼90% of this activity at the basolateral membrane. A comparable differential abundance of ZIP8 and ZIP14 in the two membranes corresponded to this functional difference (24). Inspection of the data in Figure 2, *panels C* and *D* reveal that *ATP2C1* knockdown resulted in an increase in apical ^54^Mn accumulation by 16.8%, basal accumulation by 14.4%. These data are consistent with the inference that accumulation of cytoplasmic Ca^2+^ resulting from the knockdown of this Golgi-localized pump resulted in the functional re-localization of both transporters.

Our finding that SPCA1 activity modulated Mn^2+^ uptake in hBMVECs was unexpected. Other studies have attributed SPCA1 function in reducing Mn^2+^ burden to a role in Mn^2+^ efflux (4, 33, 34). Mukhopadhyay *et. al.* correlated ^54^Mn pumping activity in isolated Golgi vesicles with SPCA1 activity; also, the Q747A hyperactive mutant supported more ^54^Mn efflux in whole cells (4). However, in this study and others that link SPCA1 activity to Mn efflux, cells were transfected to ectopically express or overexpress SPCA1 (4, 45). Also, in these studies, Mn^2+^ burden was signified by measuring cell toxicity following Mn^2+^ treatment or *via* a Mn^2+^-specific “sensor” that recorded lysosomal degradation-dependent Mn-release. Kinetic uptake and efflux assays as a direct measure of SPCA1 function in Mn trafficking were not extensively studied (4, 45). Nonetheless, SPCA1 participation in Mn efflux is not excluded by our work due to incomplete knockdown and limitations in protein overexpression. Also possible is a SPCA1 function that is cell type-dependent. In any event, to the extent that SPCA1 does contribute to Mn-trafficking, how that function interfaces with the known function of the SLC30A10 Mn-efflux transporter (ZnT10) deserves further study (12).

The observation that intracellular Ca^2+^ contributes to ZIP8 and ZIP14 trafficking underscores the complexity of the posttranslational regulation of these divalent metal-ion transporters. In addition to posttranslational modification, a variety of evidence suggests that along with other *SLC39* family members, ZIP8 and ZIP14 may form heterodimers (40, 46, 47, 48). Our Western blot analyses did give evidence of dimeric species; however, our experimental method did not explicitly interrogate whether these were homodimers or heterodimers. At a functional level, both ZIP8 and ZIP14 surface presentation is regulated by iron (38, 49). ZIP14 internalization and degradation by the proteasome was found to be ubiquitin- and deglycosylation-dependent (49), and the p53 tumor suppressor protein binds and regulates this transporter’s cell-surface localization (50). In p53 knockdown cells, ZIP14 surface occupancy and surface expression were increased. This increase in plasma membrane ZIP14 may contribute to enhanced nontransferrin–bound iron (or other metal) uptake in p53-inactivated tumor cells (50).

Both ZIP8 and ZIP14 have carboxyl-terminal domain di-leucine motifs commonly linked to retrograde retrieval from the plasma membrane. For example, dileucine motifs control the plasma membrane to Golgi trafficking of the Cu-transporter, ATP7A (51); in neurons, a dileucine signal modulates the polarized sorting of the homologous ATP7B (52). In addition, ZIP8 and ZIP14 possess tyrosine signaling markers for endocytosis that may contribute also to regulation of their surface occupancy. Within the second intracellular loop of these transporters are Src-kinase and tyrosine kinase signals, respectively (39); both kinases are modulated by intracellular calcium transients (39, 53, 54). Clearly, detailing the mechanism of ZIP8 and ZIP14 surface occupancy deserves further investigation. A detailed understanding of these processes could identify targets in the pharmacologic management of iron and manganese overload.

In conclusion, we have examined the role of SPCA1 in regulating ZIP8- and ZIP14-facilitated Mn^2+^ accumulation in hBMVECs. SPCA1 function was determined by siRNA knockdown and by overexpression of WT and a hyperactive ATPase mutant. Regulation of Mn^2+^ homeostasis in hBMVECs by SPCA1 does not appear to be functioning in the context of Mn^2+^ efflux. As determined by intracellular Ca^2+^ staining, knocking down *ATP2C1* increases intracellular Ca^2+^ levels while overexpressing SPCA1 does the opposite. Chelating intracellular Ca^2+^ decreases Mn^2+^ uptake, whereas adding exogenous Ca^2+^ to the ^54^Mn loading media enhances accumulation. Corresponding changes in ^54^Mn uptake following manipulation of cytoplasmic [Ca^2+^] were complementary changes in the plasma membrane occupancy of ZIP8 and ZIP14. Together, these data suggest that intracellular Ca^2+^ regulates surface residence of ZIP8 and ZIP14 and that the Golgi-localized import Ca-ATPase, SPCA1, can contribute to ZIP8 and ZIP14 function *via* modulation of this cytoplasmic Ca^2+^ level.

## Experimental procedures

### Cell culture

hBMVECs were a generous gift from Dr Supriya Mahajan (University at Buffalo); the generation and characteristics of this cell line have been described in detail (55, 56) and have been validated as an hBMVEC cell line (55). hBMVECs were cultured as previously described (57), reaching about 90 to 95% confluency at the time of the experiment. Experiments were performed in 3 cm, 6 cm, or 24-well tissue culture dishes.

### siRNA delivery by electroporation

*ATP2C1* expression was knocked down in hBMVECs using siGENOME human ATP2C1 siRNA SMARTpool (Dharmacon). RISC-independent siGLO transfection indicator (Dharmacon) was used as a negative control to infer transfection efficiency. The siGLO control contains a FAM-labeled fluorescent reporter to confirm appropriate nuclear localization of the siRNAs. The siRNAs were delivered *via* electroporation using the NEON transfection system (Life Technologies). Electroporation was conducted with two pulses of 1150 V for 30 ms. hBMVECs were electroporated with 125 nM siRNAs (final concentration in the culture dish) in RPMI 1640 growth media with 10% FBS (no antibiotics). Media were changed 24 h post electroporation, and cell growth, viability, and transfection efficiency (siGLO) were noted. All assays using siRNA knockdown samples were performed 24 to 92 h post electroporation.

### RT-PCR and qPCR

Total RNA was extracted from hBMVECs using the TRIzol reagent (Invitrogen) as per the manufacturer's instructions. After DNAse treatment and purification with Direct-zol RNA kit (Zymo Research), 400 ng RNA was reverse-transcribed using the iScript cDNA synthesis kit (Bio-Rad) to generate cDNA. Real-time quantitative PCR was performed using SsoAdvanced Universal SYBR Green Supermix and analyzed using a CFX96 Touch Real-Time PCR detection system (Bio-Rad). Relative expression of the target gene was normalized to *β-actin* and calculated using the ΔΔC_t_ method. Endpoint qPCR reactions were separated on a 2% agarose gel to confirm product size. The primer sequences used to amplify for each transcript were as follows: human *ATP2C1* forward: 5′-ATTGGAGCATACACTTGCCCGAGACT-3′, reverse: 5′-TGGTGCCTCTTCTGCTTGCATCA-3′; *β-actin* forward: 5′-GGGTCAGAAGGACTCCTACG-3′, reverse: 5′-GGTCTCAAACATGATCTGGG-3′.

### Plasmids and delivery by electroporation

A pCMV3 vector expressing human SPCA1 with an N-terminal FLAG tag was purchased from Sino Biological (HG14377-NF) to be used as wildtype SPCA1. The Q747A mutant was generated using site-directed mutagenesis with the KOD Xtreme Hot Start DNA Polymerase kit (Millipore Sigma) and the following primers: forward: 5′-AGACATGATCCTAGTGGATGATGATTTTGCAACCATAATGTCTGC-3′, reverse: 5′-GCAGACATTATGGTTGCAAAATCATCATCCACTAGGATCATGTCT-3′. The PCR product was used to transform *E. coli* strain DH5α. The mutant plasmid was isolated using the E.Z.N.A. Plasmid Mini Kit (Omega Bio-Tek) and analyzed *via* restriction digest with FastDigest XhoI (Thermo Scientific) on a 1% agarose gel to confirm transformation. A 400 base pair region that included the mutation was sequenced by the Roswell Park Comprehensive Cancer Center’s DNA Sequencing Core Facility to confirm the success of the mutagenesis using the sequencing primer: 5′-GTTGCAATCGCCAGTCGTC-3’. The plasmids were delivered *via* electroporation using the NEON transfection system (Life Technologies). WT SPCA1 and Q747A plasmids were electroporated at 500 ng plasmid/10^5^ cells. Electroporation was conducted with two pulses of 1150 V for 30 ms, then allowed to recover for 10 min in an Eppendorf tube prior to seeding the cells in antibiotic-free RPMI 1640 growth media with 10% FBS. Media were changed 24 h post electroporation, and cell growth and viability and transfection efficiency were noted.

### Indirect immunofluorescence

Cells were grown on sterile glass coverslips in a 6-well plate. For transfected cells, coverslips were coated with 30 μg/ml bovine collagen. Untransfected cells were seeded at 100K cells/well, and transfected cells were seeded at 200K cells/well. Cells were grown to confluency, then washed in PBS containing 1 mM CaCl_2_ and 0.5 mM MgCl_2_ (used throughout the procedure), and fixed for 10 min at RT in 3.7% paraformaldehyde and 4% sucrose in PBS. Where noted, cells were stained with Alexa Fluor 647-conjugated wheat germ agglutinin for 10 min in PBS as a cell surface marker. Cells were washed twice in PBS, blocked, and permeabilized for 1 h at room temperature using 1% BSA, 0.3 M glycine, and 0.1% Tween-20 and then incubated with primary antibody overnight at 4 °C with the following conditions: rabbit α-TGN46 Alexa Fluor 647-conjugated (Novus Biologicals, NBP-49643AF647, 1:500), α-SPCA1 (Novus Biologicals, H00027032-M01, 1:75 dilution), mouse α-FLAG antibody (GenScript, A00187, 1:500), rabbit anti-ZIP8 (Sigma-Aldrich, SAB3500598, 1:1000), or rabbit anti-ZIP14 (Sigma-Aldrich, SAB3500603, 1:1000) in 1% BSA. The following day, coverslips were washed three times in PBS and incubated with secondary antibody for 1 h in PBS with 1% BSA, using either donkey α-mouse conjugated Alexa Fluor 488 (Invitrogen, A21202, 1:1000) or donkey anti-rabbit Alexa Fluor 488 (Invitrogen, A21206, 1:1000). Coverslips were washed three times in PBS, stained with 0.7 μg/ml Hoechst for 10 min, washed, and then mounted on glass microscope slides using Prolong Gold antifade mounting media (Invitrogen). Images were obtained on the Leica TCS SP8 confocal microscope at 63X magnification with oil immersion. For ZIP8 and ZIP14 immunofluorescence, images were adjusted equally for brightness and quantified using ImageJ. Quantification was done by measuring areas around the whole cell and above the nucleus or the perinuclear region. Perinuclear regions measured were subtracted from whole-cell measurements of the same cell to acquire nonperinuclear measurements. Perinuclear and nonperinuclear regions were divided by whole-cell measurements to obtain the percent of whole-cell values. For ROI quantification, n = 10 cells in each of seven separate fields per condition were analyzed.

### ZIP14-EGFP localization

The pEGFP-N1 and pEGFP-N1-ZIP14 plasmids were a kind gift from Dr Mitchell Knutson (University of Florida). The plasmids were delivered to HEK293T/17 cells using calcium phosphate at 250 ng plasmid/10^4^ cells plated the day before transfection. Cells were allowed to recover for 24 h, then media were changed 24 h posttransfection, and cell viability and transfection efficiency were monitored.

For fluorescence imaging, transfected cells were grown on collagen-coated (30 μg/ml) sterile glass coverslips in a 6-well plate at a density of 10^4^ cells/well. Cells were grown to confluency in Dulbecco's modified Eagle's medium (DMEM) minus Ca^2+^, plus serum for 2 days prior to the start of the assay. Cells were then treated with 2 mM Ca^2+^ or 1 μg/ml LPS for 2 h in DMEM minus Ca^2+^, minus serum. After treatment, cells were washed twice in PBS containing 0.5 mM MgCl_2_, minus Ca^2+^ and fixed for 10 min in 3.7% paraformaldehyde in PBS. Cells were counterstained with Alexa Fluor 647-conjugated wheat germ agglutinin and 0.7 μg/ml Hoechst 33342 for 10 min in PBS, washed, and mounted on glass microscope slides using ProlongTM Gold antifade mounting media (Invitrogen). Images were obtained on the Leica TCS SP8 confocal microscope at 63X magnification with oil immersion. Quantification was done using ImageJ software. The three channels were split, and the green channel was used for quantification. The line tool was used to draw a line from the outside of the cell, through the membrane, to inside the cell. Every line was the same size, 0.682 μm. Four regions from three different cells were quantified for each condition, therefore n =12. The resulting histograms for the plasma membrane peak ± 0.2 μm were subjected to nonlinear regression fit to a Gaussian model, and the 95% confidence intervals were calculated and plotted.

### ^54^Mn accumulation and ^54^Mn efflux assays in monolayers

For ^54^Mn accumulation assays, hBMVEC monolayers were loaded with physiological concentrations (200 nM) of ^54^Mn^2+^ (PerkinElmer) in RPMI 1640 plus serum growth media. Reactions were terminated with ice-cold quench buffer as previously described (58), then lysed with RIPA buffer (25 mM Tris, 150 mM NaCl, 1% NP-40, 1% Na-deoxycholate, 0.1% SDS). For ^54^Mn efflux assays, hBMVEC monolayers were loaded with 200 nM ^54^Mn^2+^ for 18 h. Cells were washed once with RPMI 1640 (minus serum, 5 μg/ml human insulin, 30 nM Na-selenite) and twice with 250 μM sodium citrate. ^54^Mn^2+^ efflux was monitored in RPMI 1640 plus serum growth media for 3 h, as described. Efflux was quenched as above, and cells lysed with RIPA buffer. Lysates were analyzed for ^54^Mn and protein content. ^54^Mn counts (LKB Wallac CompuGamma) were normalized to protein concentration. Protein was quantified by BCA assay (Thermo Scientific).

### Apical and basolateral ^54^Mn accumulation in transwells

hBMVECs grown in transwells were loaded for 24 h with physiological concentrations (200 nM) of ^54^Mn^2+^ (PerkinElmer) in RPMI 1640 plus serum growth media when loaded from the apical chamber and RPMI 1640 minus serum growth media when loaded from the basal chamber. Reactions were terminated with ice-cold quench buffer as previously described (57), then lysed with RIPA buffer (25 mM Tris, 150 mM NaCl, 1% NP-40, 1% Na-deoxycholate, 0.1% SDS). Lysates were analyzed for ^54^Mn and protein content. ^54^Mn counts (LKB Wallac CompuGamma) were normalized to protein concentration quantified by BCA assay (Thermo Scientific, Rockford, IL).

### Oregon Green BAPTA 488 (OGB1) intracellular Ca^2+^ staining

For OGB1 staining in ATP2C1 knockdown cells, hBMVECs were electroporated with mock, siATP2C1, or Q747A SPCA1-FLAG as previously described. For OGB1 in untransfected hBMVECs, cells were grown in RPMI 1640 minus Ca^2+^ media plus 10% FBS for 2 days prior to 1.5 h pretreatment in RPMI 1640 minus Ca^2+^, minus serum. Where indicated, cells were treated with 10 μM BAPTA-AM or 2 mM CaCl_2_ during the pretreatment. hBMVECs were incubated with 1 μM OGB1 (or no dye control) for 1 h, washed twice in PBS, then imaged at 20X magnification on a Bio-Rad ZOE imager. Images were quantified with ImageJ using n ≥ 5 ROIs per image, and the intensities were background corrected using the no OGB1 stain control and normalized to the untreated control or mock samples.

### Cell-surface biotinylation

Membrane proteins were separated from hBMVECs as previously described (59). Briefly, cells grown in 6-well plates were washed twice with PBS containing 1 mM CaCl_2_ and 0.5 mM MgCl_2_ (used for entire biotinylation protocol), then treated with 0.5 mg/ml EZ-Link Sulfo-NHS-SS-Biotin (ThermoFisher) for 2 h at 4 °C. Then, cells were washed twice with PBS containing 0.1% BSA and twice with PBS. Cells were lysed by scraping in ice-cold RIPA buffer (25 mM Tris, 150 mM NaCl, 1% NP-40, 1% Na-deoxycholate, 0.1% SDS, pH 7.4) supplemented with 4x Halt protease inhibitor cocktail (ThermoFisher) and incubation on ice for 15 min. The cell suspension was then centrifuged at 10,000*g* for 10 min at 4 °C, and the supernatant (input fraction) was collected and loaded onto a NeutrAvidin Agarose column overnight at 4 °C. Following collection of the flow-through (unbound fraction), columns were washed with RIPA buffer containing 4x protease inhibitors and eluted at 50 °C in 6x SDS-loading buffer, 150 mM DTT (bound fraction). The input and unbound fraction protein content was quantified, and equal amounts of protein were loaded for Western blotting. All of the elution from the bound fraction was used for SDS-PAGE followed by western blotting.

### Western blotting

Lysate samples (see RIPA + 4x protease inhibitor recipe above) were denatured at 37 °C for 30 min (20 μg total protein/lane) and fractionated on 4 to 15% SDS-polyacrylamide gradient gels, followed by transfer to a PVDF membrane. Membranes were blocked in TBST (Tris-buffered saline with 0.05% Tween-20) containing 5% milk at RT for 1 h. Primary antibodies were diluted in 1% milk-TBST as follows: 1:2500 mouse α-SPCA1 antibody (Novus Biologicals, H00027032-M01), 1:2000 to 2500 rabbit α-ZIP8 antibody (Sigma-Aldrich, SAB3500598), 1:2500 rabbit α-ZIP14 antibody (Sigma-Aldrich, SAB3500603), and 1:5000 dilution of rabbit α-β-actin antibody (Cell Signaling Technologies, 4970S). Validation of the specificity of the antibodies for ZIP8 and ZIP14 was provided by two approaches as described (24). First, the bands identified as ZIP8 and ZIP14 were knocked down in hBMVECs expressing cognate siRNA oligonucleotides. Second, staining of these specific bands was blocked following preincubation with antigen-specific peptides. Blots were incubated with primary antibody at 4 °C overnight. After washing, membranes were incubated at RT for 1 h with secondary donkey α-mouse horseradish peroxidase-conjugated antibody (Novus Biologicals, NBP2-30347H, 1:5000 dilution) or 1:7500 dilution of goat α-rabbit horseradish peroxidase-conjugated antibody (Novus Biologicals, NBP2-30348H) in TBST containing 3% milk. Immunocomplexes were visualized using SuperSignal West Dura Extended Duration Substrate (Thermo Scientific) on a ChemiDoc Imager and images processed using Image Lab software (Bio-Rad).

### Statistical analysis

Statistical analyses were performed using Prism 8.0 or 9.0 (GraphPad Software). Data are presented as mean ± SD. Unpaired *t* tests were used when comparisons were made between two conditions (one variable) from the same time point. Comparisons of multiple samples were made using one-way ANOVA statistical analyses in conjunction with Tukey’s multiple comparison tests. In the text, “n =” refers to the number of total (biologic and technical) replicates used to derive the stated quantity.

## Data availability

All relevant data are contained within this article.

## Conflict of interest

The authors declare that they have no conflicts of interest with the contents of this article.
